# Medication Adherence Monitoring Using Alternative Sample Matrices: Bridging the Gap Between Analytical Validation and Clinical Interpretation

**DOI:** 10.1097/FTD.0000000000001220

**Published:** 2024-06-07

**Authors:** Tanja R. Zijp, Zamrotul Izzah, Daan J. Touw, Job F. M. van Boven

**Affiliations:** * Department of Clinical Pharmacy and Pharmacology, University Medical Center Groningen, University of Groningen, Groningen, the Netherlands;; † Department of Pharmaceutical Analysis, Groningen Research Institute of Pharmacy, University of Groningen, Groningen, the Netherlands;; ‡ Department of Pharmacy Practice, Faculty of Pharmacy, Universitas Airlangga, Surabaya, Indonesia; and; § Medication Adherence Expertise Center of the Northern Netherlands (MAECON), Groningen, the Netherlands

***To the Editor:***

With great interest, we have read the study of Jacobs et al.^1^ The authors performed a systematic review on adherence monitoring in sample matrices using mass spectrometry (MS) in the context of clinical toxicology. Notably, using direct adherence monitoring with biological matrices not only can qualitative questions be answered (whether the patient is abstinent of substance use/drugs of abuse), but quantitative answers may also be given (whether the amount of medication taken is in line with the prescribed amount). In their systematic review, the authors identified 37 articles that met the inclusion criteria and used urine, dried blood spots (DBS), oral fluid, exhaled breath, and scalp hair as sample matrices with venous blood (serum or plasma) as the reference material. The advantages and disadvantages of the applied methods are evaluated in each study. While this evaluation primarily focused on bioanalytical MS procedures, the authors concluded that apart from the challenges regarding the particular matrices used, the most difficult part was data interpretation as the basis for clinical decision making. For most matrices, reference concentration ranges and cutoffs and the pharmacokinetic data of the target substances in these matrices are unavailable. Therefore, the use of MS for adherence evaluation may remain limited. However, there is much knowledge to be gained by research performed *after* analytical detection but *before* clinical interpretation. Although only superficially discussed by the authors, we believe that this crucial yet complex step on the road to clinical implementation deserves more detailed attention in validation studies.

**FIGURE 1.**
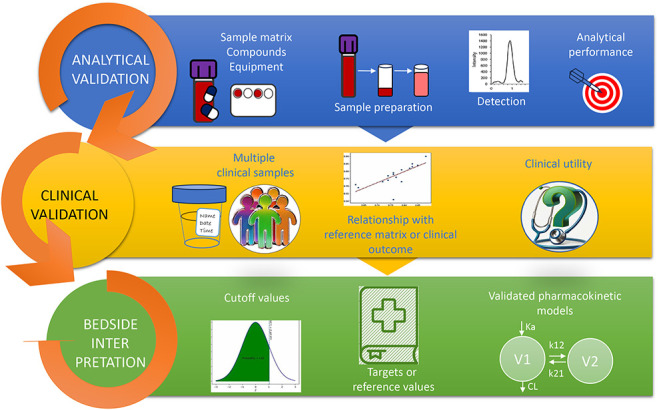
Proposed steps of validation of a bioanalytical method for clinical application. First, the method undergoes analytical validation including the choice of matrix, compound and equipment, sample preparation, and testing the reliability of the analytical results and method performance. Second, for clinical validation, the method is tested in multiple clinical samples from a predefined context and population, where results are correlated with a reference matrix or clinical outcome, and its clinical utility is assessed (eg, the question is answered if this method can be used for its proposed use). Third, to interpret the results of an individual patient and guide clinical decisions, cutoff values, target values, reference values, and/or validated pharmacokinetic models should be used.

After analytical validation to accurately measure the targeted drug for adherence, we believe that the next step should be clinical validation (Fig. 1). Clinical validation investigates whether the method is suitable for the “real-world” context and population, which are typically different from controlled laboratory settings. These differences entail limitations in patient sampling (eg, DBS sampling errors due to tremors), analytical errors (eg, interference by metabolites), and biological and pharmacokinetic variability (eg, circadian rhythms and sampling times), and how these factors affect the results should be evaluated.

However, only a limited number of guidelines and quality standards describe how target drug measurements should be validated for use in clinical practice. Measurements should be performed on target drug-positive clinical samples. Regarding DBS, a minimum of patient and sample numbers has been described by Capiau et al^2^ with 40 patient samples or ≥25 patients with multiple samples being required for the clinical validation. With these samples, cross-validation should be performed for each matrix, either with a reference method (eg, determination in blood or plasma), another adherence measurement method (eg, electronic monitoring^3^), or a clinical outcome (eg, blood pressure for antihypertensives^4^). We believe that the feasibility of this method for clinical use needs to be studied. Without any form of clinical validation, the analytical results should be interpreted with caution.

In line with our proposed requirement for a clinical validation step, we have previously performed a systematic scoping review to identify studies reporting a bioanalytical method for adherence or therapeutic drug monitoring that included a form of clinical validation in saliva, DBS, and hair over the course of 20 years (2000–2020).^5^ Following this review, our recommendations are: (1) “All analytical methods should be clinically validated to prove their applicability for routine care; (2) a clinical validation for emerging methods should include real patient samples acquired from a sufficient number of patients in addition to the performance of a cross-validation with a previously validated method or with valid clinical outcomes; (3) further reviews should consider which other parameters should be used for evaluation and/or standardisation; and (4) further research should address the translation from clinical trials to routine patient care.”^5^

Following clinical validation, cutoff and/or reference values based on the measured clinical samples should be made available to allow clinical interpretation. Ideally, pharmacokinetic models could be used to describe the distribution of the target drug in the matrix of interest, which could further personalize the interpretation of the results. However, this pharmacokinetic model needs to be validated.

Because medication adherence is a prevalent and significant problem, there is an increasing trend in the possibility of directly monitoring medication adherence using emerging bioanalytical methods. In light of the publication by Jacobs et al, we look forward to new studies performed in such a way that they support actual implementation in clinical practice.
